# EPOS-OHCA: Early Predictors of Outcome and Survival after non-traumatic Out-of-Hospital Cardiac Arrest

**DOI:** 10.1016/j.resplu.2024.100728

**Published:** 2024-07-24

**Authors:** Julian Kreutz, Nikolaos Patsalis, Charlotte Müller, Georgios Chatzis, Styliani Syntila, Kiarash Sassani, Susanne Betz, Bernhard Schieffer, Birgit Markus

**Affiliations:** a Philipps University of Marburg, Germany; b University Hospital of Marburg, Department of Cardiology, Angiology, and Intensive Care Medicine, Germany; c University Hospital of Marburg, Center for Emergency Medicine, Germany

**Keywords:** Out-of-hospital cardiac arrest (OHCA), Post-resuscitation management, Prognostic parameters, Outcome

## Abstract

**Background:**

Post-cardiac arrest syndrome (PCAS) after out-of-hospital cardiac arrest (OHCA) poses significant challenges due to its complex pathomechanisms involving inflammation, ischemia, and reperfusion injury. The identification of early available prognostic indicators is essential for optimizing therapeutic decisions and improving patient outcomes.

**Methods:**

In this retrospective single-center study, we analyzed real-world data from 463 OHCA patients with either prehospital or in-hospital return of spontaneous circulation (ROSC), treated at the Cardiac Arrest Center of the University Hospital of Marburg (MCAC) from January 2018 to December 2022. We evaluated demographic, prehospital, and clinical variables, including initial rhythms, resuscitation details, and early laboratory results. Statistical analyses included logistic regression to identify predictors of survival and neurological outcomes.

**Results:**

Overall, 46.9% (n = 217) of patients survived to discharge, with 70.1% (n = 152) achieving favorable neurological status (CPC 1 or 2). Age, initial shockable rhythm, resuscitation time to return of spontaneous circulation (ROSC), and early laboratory parameters like lactate, C-reactive protein, and glomerular filtration rate were identified as independent and combined *Early Predictors of Outcome and Survival* (EPOS), with high significant predictive value for survival (AUC 0.86 [95% CI 0.82–0.89]) and favorable neurological outcome (AUC 0.84 [95% CI 0.80–0.88]).

**Conclusion:**

Integration of EPOS into clinical procedures may significantly improve clinical decision making and thus patient prognosis in the early time-crucial period after OHCA. However, further validation in other patient cohorts is needed.

## Introduction

One of the biggest challenges in intensive care medicine is the time-critical determination of the most appropriate therapy for critically ill patients. In out-of-hospital cardiac arrest (OHCA) patients, the outcome ultimately depends on resuscitation-related factors such as the initial heart rhythm and the duration of cardiopulmonary resuscitation (CPR). However, in these patients timely and accurate decision-making is critical due to the post-cardiac arrest syndrome (PCAS) that occurs after OHCA. In particular, PCAS is a highly complex and multifaceted condition characterized by inflammation, ischemia, and reperfusion injury, causing damage that is often irreversible over time.^1^, ^2^, ^3^

Thus, a key aspect of improving patient outcomes is the identification of early prognostic indicators that can guide clinical decision-making and tailor interventions to individual patient needs. The most optimal, highly specified, and specialized therapy should be offered according to clinical, ethical, and ultimately, socio-economic considerations. Nevertheless, the definition of therapy limits based on the predicted outcome and quality of life also appears mandatory in this context.^4^, ^5^, ^6^ However, identifying treatment options and limitations is often difficult because there are few early, common, and therefore valid factors that can be used to make a reliable assessment.^7^, ^8^, ^9^, ^10^, ^11^ Nowadays, in addition to the universally valid critical care scores such as *Therapeutic Interventions Scoring System* (TISS), *Simplified Acute Physiology Score* (SAPS) and *Sequential Organ Failure Assessment* (SOFA), new prognostic scoring systems such as *Post-Cardiac Arrest Syndrome for Therapeutic Hypothermia* (CAST) and *Pittsburgh Cardiac Arrest Category* (PCAC) have been defined. They all consider various factors and endpoints with a particular focus on neurological outcome at different time points of therapy after OHCA.^12^, ^13^ The TISS and SAPS scores are primarily used to assess organ function and the extent of nursing care on the intensive care unit (ICU). In contrast, the CAST score and its simplified version (rCAST) are used early after OHCA for prognostic evaluation, originally to guide the initiation of targeted temperature management (TTM). Both, the CAST and rCAST scores have shown high accuracy in predicting neurological prognosis and they are superior in validity to PCAC score.^12^, ^14^, ^15^, ^16^ However, it is not yet widely used for basic treatment decisions, as the prognostic significance varies due to a non-generalized observation approach limited by rare parameters.

To support treatment decision-making in a time-critical situation, early available and easily applicable predictors of survival and neurological outcome were identified from five years of real-world data of post-resuscitation management at the Marburg University Hospital's Cardiac Arrest Center (MCAC) in Germany.

## Materials and methods

### Setting

This retrospective, single-center study analyses data from patients admitted to the MCAC after OHCA from January 2018 to December 2022. This includes pre-hospital data from emergency medical services (EMS) and inpatient care data from local databases. From 2018 to 2020, data was only collected from patients referred to the MCAC by EMS in one county. In 2021 and 2022, patients from surrounding counties were also included, with all EMS using uniform standards and equipment.

### Inclusion

Patients were included if they were ≥ 18 years old, had experienced non-traumatic cardiac arrest, received pre-hospital resuscitation by EMS, and achieved return of spontaneous circulation (ROSC). The analysis included the entire cohort, distinguishing between “survivors” (discharged alive) and “non-survivors” (died during hospitalization).

### Study design

The rCAST score was calculated for patients with complete data sets of the parameters included in the score: initial cardiac arrest rhythm, time to ROSC, pH value and lactate from arterial blood gas analyses, and Glasgow Coma Scale (GCS) motor score. Neurological outcome was assessed using Cerebral Performance Category (CPC) and Modified Rankin Scale (mRS) at discharge, with CPC status 1/2 or mRS 0–3 defined as a “favorable” neurological outcome and CPC status 3–5 or mRS ≥ 4 defined as “poor” neurological outcome. For each patient, the CPC/mRS score was determined by two independent intensive care physicians at the time of discharge from the hospital.

### Analysis

IBM SPSS Statistics (v29), GraphPad Prism (v10) and Stata (v16) were used for statistical analysis. Continuous variables were summarized as mean ± SD or median and interquartile range (IQR). Independent samples t-tests with Satterthwaite correction for unequal variances compared continuous variables between “survivors” and “non-survivors”. Chi-squared tests with Yates correction were used to analyze categorical variables and the Mann-Whitney *U* test was used for non-parametric data, directly comparing medians. The significance level was set at 0.05 for all tests. Logistic regression models were performed to identify independent predictors of early outcome among variables that showed statistically significant differences in the preliminary univariate analysis. Stata software was used to calculate the area under the receiver operating characteristic (ROC) curve (AUC) for each logistic regression model. DeLong test was used to compare differences between AUC of ROC curves. The AUC values, together with the corresponding ROC curves, were generated to assess the discriminatory ability of the models to effectively discriminate between the outcome of “survivors” and “non-survivors” based on the predictors and rCAST score. For each identified significant predictor, cut-off values were determined using Youden's index.

### Ethics

The retrospective analysis was approved by the local Ethics Committee of the Philipps University of Marburg in accordance with the Declaration of Helsinki (ek_mr_14072021).

## Results

564 patients with OHCA were referred to the MCAC. After excluding 101 patients due to incomplete data or failure to meet the inclusion criteria (Supplementary Table 1), data from 463 patients could be analyzed. The reasons for the resuscitation event within the included patient cohort are listed in Supplementary Table 2. 46.9% of the patients (n = 217) survived to be discharged home or to rehabilitation, while 53.1% (n = 246) died in hospital. Of the “non-survivors”, 7.3% (n = 18) died in the emergency department. 38.2% (n = 94) died within 24 h on the ICU due to the severity of their condition and 54.5% (n = 134) died more than 24 h after admission.

In this context, it should be mentioned that this time (24 h) is not decisive for the decision to withdraw life-sustaining treatment (WLST). The decision to WLST is usually made more than 72 h after the relevant diagnosis unless earlier intervention is required (e.g. due to cerebral perfusion failure).

Of the “survivors”, 70.1% (n = 152) had a favorable neurological outcome (CPC 1/2 or mRS 0–3), which improved to 77.2% when excluding patients with pre-resuscitation CPC 3/4 or mRS 4–5. Of the 71 patients who underwent extracorporeal CPR (eCPR), 16.9% (n = 12) survived to discharge, with 58.3% (n = 7) having a favorable outcome.

### Demographics and comorbidities

Demographic and comorbidity analysis (Table 1) revealed that “non-survivors” were significantly older (67.1 ± 15.2 vs. 64.1 ± 13.0 years, *p* = 0.02), less often male (66.7% vs. 78.8%, *p* = 0.004), had a higher body mass index (BMI, 29.3 ± 7.0 vs. 27.7 ± 4.2, *p* = 0.02), and were more likely to have pre-existing chronic renal failure (KDIGO ≥ stage 3) than “survivors” (12.8% vs. 7.0%, *p* = 0.05).

**Table 1:** Demographics and comorbidities of “non-survivors” vs. “survivors”.

**Demographics and comorbidities**	**n=**	**non-survivors**	**survivors**	**p-value**
Number of patients^1^	463	246 (53.1)	217 (46.9)	
Age (years)^2^	463	67.1 (±15.2)	64.1 (±13.0)	**0.02**
Male sex^1^	463	164 (66.7)	171 (78.8)	**0.004**
BMI (kg/m^2^)^2^	308	29.3 (±7.0)	27.7 (±4.2)	**0.02**
MI in the past/ CHD^1^	431	42 (19.3)	40 (18.8)	0.90
Vitium of aortic/mitral valve (grade 2/3)^1^	431	16 (7.5)	16 (7.5)	0.95
Heart failure ≥ NYHA 3^1^	431	12 (5.5)	15 (7.0)	0.51
Atrial fibrillation^1^	431	30 (13.8)	25 (11.7)	0.53
Pacemaker ^1^	431	9 (4.1)	5 (2.3)	0.30
Arterial hypertension^1^	431	112 (51.4)	107 (50.2)	0.81
Hyperlipidemia^1^	431	30 (13.8)	39 (18.3)	0.20
Diabetes mellitus^1^	431	42 (19.3)	35 (16.4)	0.44
Nicotine abuse (>5py)^1^	431	46 (21.1)	57 (26.8)	0.17
Alcohol abuse^1^	431	13 (6.0)	13 (6.1)	0.95
Chronic renal failure (KDIGO ≥ stage 3)^1^	431	28 (12.8)	15 (7.0)	**0.05**
Renal replacement therapy^1^	463	11 (4.5)	5 (2.3)	0.20
COPD ≥ GOLD 2^1^	431	22 (10.1)	18 (8.5)	0.56
Bronchial asthma^1^	431	6 (2.8)	5 (2.3)	0.79
OSAS^1^	431	10 (4.6)	8 (3.8)	0.67
Pre-existing tracheostoma^1^	431	4 (1.6)	3 (1.4)	0.83
Cerebral Stroke^1^	431	19 (8.7)	17 (8.0)	0.78
Thrombosis/ PAE^1^	431	6 (2.8)	4 (1.9)	0.55
Malignant disease^1^	431	23 (10.6)	16 (7.5)	0.20
Peripheral arterial disease (stage ≥ 2)^1^	431	11 (5.0)	8 (3.8)	0.52
Carotid artery stenosis^1^	431	10 (4.6)	8 (3.8)	0.67
Hypo- or hyperthyroidism^1^	431	17 (7.8)	12 (5.6)	0.37

Abbreviations: BMI: body mass index, MI: myocardial infarction, CHD: coronary heart disease, COPD: chronic obstructive pulmonary disease, OSAS: obstructive sleep apnea syndrome, PAE: pulmonary artery embolism, py: pack years.^1^: n (%); ^2^: Mean (SD).

### Pre-hospital parameters of resuscitation and baseline laboratory parameters

Pre-hospital rhythm analysis showed that “non-survivors” had a lower incidence of shockable rhythm (22.0% vs. 64.1%, p < 0.001) and a higher incidence of asystole (45.9% vs. 12.9%, p < 0.001) compared to “survivors”. “Non-survivors” were less likely to have witnessed cardiac arrest (65.9% vs. 73.3%, *p* = 0.08) and more likely to require more than 20 min of resuscitation to achieve ROSC (66.7% vs. 40.1%, p < 0.001). The median resuscitation time to ROSC (minutes) was 26.00 (IQR 15.00–64.50) for non-survivors and 15.00 (IQR 8.00–27.50) for survivors, p < 0.001. The use of chest compression devices was higher in “non-survivors” (29.7% vs. 10.2%, p < 0.001) and these patients had a significantly longer resuscitation time, which may partly explain the difference in survival rates. No significant differences were observed between “non-survivors” and “survivors” in prehospital airway management, including the frequency of intubation attempts (23.1% vs. 16.6%, *p* = 0.11) and the use of video laryngoscope (32.9% vs. 24.4%, *p* = 0.06). Similarly, systemic lysis therapy during resuscitation (3.0% vs. 2.9%, *p* = 0.51) and the use of intraosseous access (16.7% vs. 11.1%, *p* = 0.11) did not differ significantly between groups.

However, “non-survivors” had lower arterial pH (7.12 [IQR 6.95–7.26] vs. 7.27 [IQR 7.19–7.34], p < 0.001), higher arterial lactate levels (10.55 [IQR 6.01–15.00] vs. 4.1 [IQR 2.4–6.68], p < 0.001), higher C-reactive protein (CRP) levels (10.00 [IQR 2.50–39.00] vs. 3.95 [IQR 1.73–9.35], p < 0.001) and lower glomerular filtration rate (GFR) (45.00 [IQR 32.00–56.00] vs. 61.0 [IQR 45.0–75.5], p < 0.001) within one hour after admission to the MCAC compared to “survivors” (Table 2). The median time from the time EMS arrived at the scene to the time transport to the hospital for further care was 48.0 min for “survivors” (IQR 38.0–56.0) and 53.5 min for “non-survivors” (IQR 41.0–65.0).

**Table 2:** **Pre-hospital resuscitation-associated and baseline laboratory parameters of “non-survivors” vs. “survivors”.** Baseline laboratory parameters were taken within the first hour after hospital admission.

	**n=**	**non-survivors**	**survivors**	**p-value**
**Resuscitation-associated parameters**				
Initial shockable rhythm (VF/VT)^1^	463	54 (22.0)	139 (64.1)	**<0.001**
Initial rhythm: Asystole^1^	463	113 (45.9)	28 (12.9)	**<0.001**
Initial rhythm: PEA^1^	463	79 (32.1)	50 (23.0)	**0.03**
Witnessed cardiac arrest^1^	463	162 (65.9)	159 (73.3)	0.08
Bystander CPR^1^	463	139 (56.5)	133 (61.3)	0.30
Resuscitation time > 20 minutes^1^	463	164 (66.7)	87 (40.1)	**<0.001**
Resuscitation time until ROSC (min)^3^	461	26.00 (15.00 – 64.50)	15.00 (8.00 – 27.50)	**<0.001**
Mechanical CPR (chest compression device)^1^	463	73 (29.7)	22 (10.2)	**<0.001**
Prehospital airway management: more than 1 intubation attempt^1^	409	50 (23.1)	32 (16.6)	0.11
Prehospital airway management: use of video laryngoscope^1^	409	71 (32.9)	47 (24.4)	0.06
Systemic lysis therapy during resuscitation^1^	452	7 (3.0)	6 (2.9)	0.51
Intraosseous (i.o.) access^1^	463	41 (16.7)	24 (11.1)	0.11
**Baseline laboratory parameters (≤1 h after admission)**				
pH (arterial)^3^	438	7.12 (6.95 – 7.26)	7.27 (7.19 – 7.34)	**<0.001**
Lactate (mmol/l, arterial)^3^	438	10.55 (6.01 – 15.00)	4.10 (2.40 – 6.68)	**<0.001**
CRP (mg/l)^3^	445	10.00 (2.50 – 39.00)	3.95 (1.73 – 9.53)	**<0.001**
GFR (ml/min)^3^	430	45.00 (32.00 – 56.00)	61.00 (45.00 – 75.50)	**<0.001**

Abbreviations: VF: ventricular fibrillation, VT: ventricular tachycardia, PEA: Pulseless electrical activity, ROSC: return of spontaneous circulation, CRP: C-reactive protein, GFR: glomerular filtration rate. ^1^: n (%), ^3:^ Median (IQR).

The study found that “non-survivors” received significantly higher dosages of epinephrine during resuscitation than survivors (4.00 [IQR 2.00–8.00] vs. 1.00 [IQR 0.00–4.00], p < 0.001). Higher doses in “non-survivors” correlated with prolonged resuscitation and fewer initial shockable rhythms, consistent with guidelines. Therefore, epinephrine dose was excluded from further regression analysis to identify early predictors.

### Identification of early available predictors of outcome

**Fig. 1:**
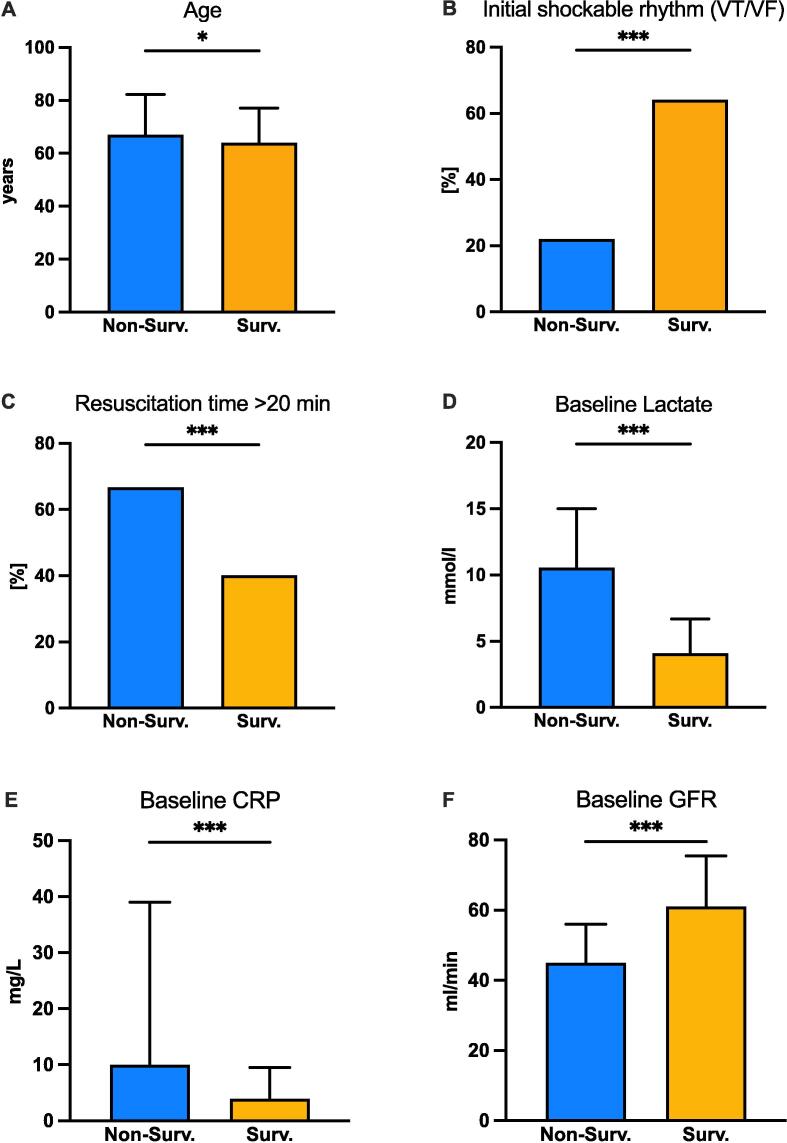
**Significant predictors identified by multiple regression analysis − defined as *Early Predictors of Survival and Outcome* (EPOS) after OHCA.** A: age (years), Mean (SD); B: initial shockable rhythm (%); C: resuscitation time > 20 min (%), D: baseline lactate level (mmol/l), Median (IQR); E: baseline CRP level (mg/l), Median (IQR); F: initial GFR level (ml/min), Median (IQR). Abbreviations: VT: ventricular tachycardia, VF: ventricular fibrillation, CRP: C-reactive protein, GFR: glomerular filtration rate, Non-Surv.: “non-survivors”, Surv.: “survivors”. (*p < 0.05, ***p < 0.001).

To identify early independent predictors of outcome, the significant variables from the univariate analysis were analyzed using multiple regression, with two models shown in the Appendix (Supplementary Table 3). BMI was excluded because of its limited power and availability. Age significantly predicted survival, whereas gender, chronic renal insufficiency, and previous cardiac arrest did not. Initial shockable rhythm and resuscitation of less than 20 min strongly correlated with survival. The use of chest compression devices showed a negative but not significant correlation. In a model excluding lactate, the initial pH correlated strongly with lactate and survival (OR 13.01 [95% CI 2.97–56.91], p < 0.001). Lactate levels from arterial blood gas analysis, higher baseline CRP values (natural log), and lower GFR values were negatively associated with survival. The six significant predictors finally identified are shown in Fig. 1.

To obtain the most suitable results, the data were further analyzed and statistically evaluated. The above-mentioned six variables were again included in a logistic regression analysis. In this logistic regression model, the categorical variable of resuscitation time was replaced by the continuous variable of time (min) to ROSC and values were very stable when GFR was adjusted for the presence of CKD (data not shown). Table 3 shows the model and the significant E*arly Predictors of Outcome and Survival* (EPOS) after OHCA with an AUC of 0.86 for survival, indicating strong predictive power.

**Table 3:** Early Predictors of Outcome and Survival (EPOS) after OHCA.

**Number of valid data (n = )**	**416**	
Pseudo-R^2^ (Cox&Snell; Nagelkerke)	0.37; 0.49	
AUC [95% CI]	0.86 [0.82 – 0.89]	
	**OR (95% CI)**	**p-value**
**Demographics and comorbidities**		
Age (years)	0.97 (0.95 – 0.99)	0.001
**Resuscitation-associated parameters**		
Initial shockable rhythm (VF/VT)	2.98 (1.76 – 5.05)	<0.001
Resuscitation time until ROSC (min)	0.99 (0.98 – 1.00)	0.005
**Baseline laboratory parameters (≤1 h after admission)**		
Lactate (from arterial blood gas analysis)	0.85 (0.79 – 0.90)	<0.001
CRP	0.79 (0.67 – 0.94)	0.008
GFR	1.01 (1.00 – 1.02)	0.02

Logistic regression model adjusting for age, initial shockable rhythm, resuscitation time until ROSC, baseline levels of lactate, CRP, and GFR. Abbreviations: VT: ventricular tachycardia, VF: ventricular fibrillation, ROSC: return of spontaneous circulation, CRP: C-reactive protein, GFR: glomerular filtration rate.

Cut-off values for predictors within the EPOS model essential for survival prediction were determined using Youden’s index (Supplementary Table 4). These values include age at 67.5 years; time from resuscitation initiation to ROSC at 18.5 min; lactate concentration within one hour after admission at 8.2 mmol/L; CRP at 12.0 mg/L; and GFR at 60.5 mL/min.

In the study, the rCAST score was calculated for 438 patients. Patients with a favorable neurological outcome (CPC 1/2) had a median rCAST score of 8.00 (IQR 5.13–10.38), which was significantly different from patients with a poor neurological outcome (CPC 3–5), who had a median rCAST score of 12.00 (IQR 9.50–15.00) (p < 0.001). In patients with poor neurological outcome, the median score was 9.50 (IQR 8.00–12.00) for CPC 3/4 and 13.00 (IQR 10.00–15.50) for CPC 5 (death).

**Fig. 2:**
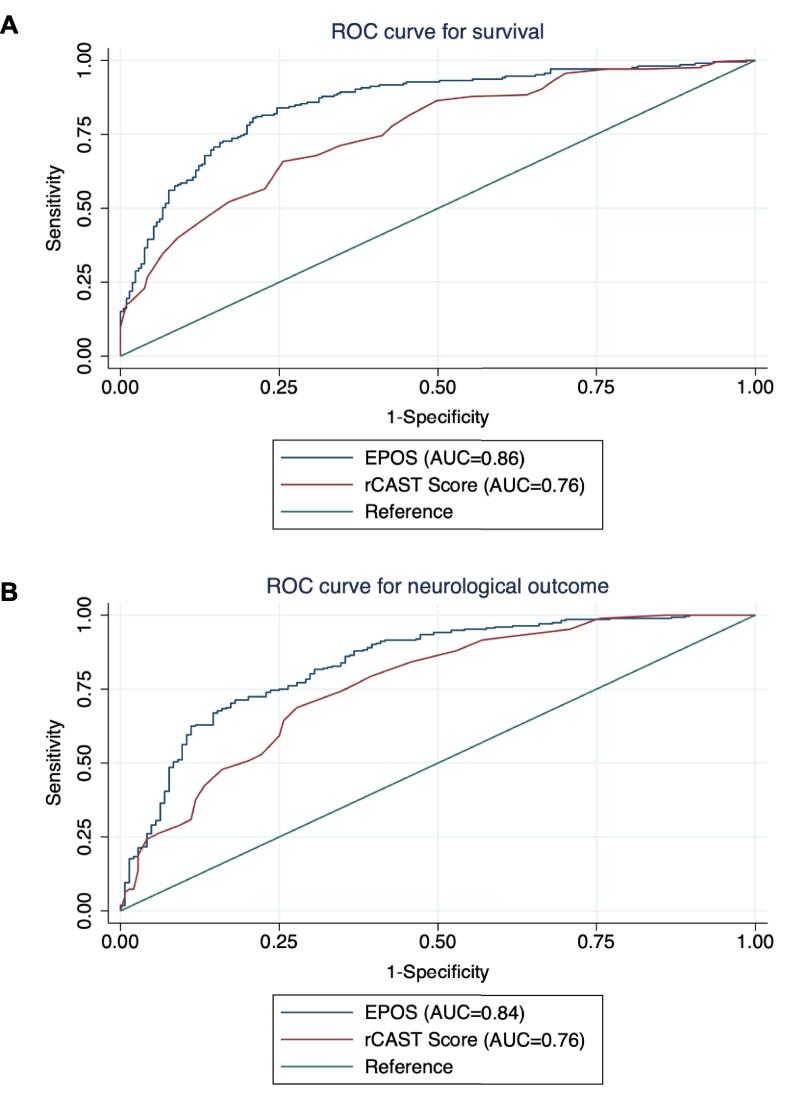
**Comparative analysis of the predictive power of the EPOS model and the rCAST score.** Panel A shows ROC curves for survival comparing the EPOS model (blue curve, AUC 0.86 [95% CI 0.82–0.89]) with the rCAST score (red curve, AUC 0.76 [95% CI 0.72–0.81]). Panel B shows ROC curves for neurological outcomes of the EPOS model (blue curve, AUC 0.84 [95% CI 0.80–0.88] vs. the rCAST score (red curve, AUC 0.76 [95% CI 0.72–0.81]). (For interpretation of the references to colour in this figure legend, the reader is referred to the web version of this article.)

To evaluate the EPOS model against the established rCAST score, ROC curves were compared for both, survival, and neurological outcomes (Fig. 2). The study showed that the EPOS model had better predictive accuracy (DeLong Test: p < 0.001) for overall survival (AUC 0.86) than the rCAST score (AUC 0.76). Similarly, the EPOS model outperformed the rCAST score for neurological outcome with an AUC of 0.84 compared to 0.76 (DeLong Test: *p* = 0.001).

## Discussion

To further support treatment decisions, we here identified a combination of *Early Predictors of Outcome and Survival after OHCA* (EPOS-OHCA). A comprehensive analysis of various patient and resuscitation-related data has identified six parameters that, when used in combination, seem to be superior to known and established scores such as the rCAST score. In detail, EPOS contains demographic data, resuscitation-associated parameters, and early laboratory parameters. Using multiple regression analysis, age, initial shockable rhythm, and time to ROSC were shown to be independent predictors of survival, similar to the current literature.^17^, ^18^, ^19^ Interestingly, our study also found that early lactate levels, as well as CRP and GFR, were independent predictors when measured within the first hour after hospital admission. Except for lactate, these markers have only been investigated to a limited extent in previous studies. However, they should not be neglected as they reflect inflammation, quality of perfusion, and organ function, and are therefore important indicators of the severity of PCAS after OHCA.^20^, ^21^

In addition, the results of this study once again underline the prognostic importance of the quality of renal function and the impact of renal failure on treatment outcomes.^22^, ^23^

Systemic inflammation, primarily driven by ischemia–reperfusion injury, is a critical pathophysiological disturbance in PCAS following OHCA. Recent studies have repeatedly investigated the role of inflammatory markers as prognostic biomarkers in PCAS, which is often described as “sepsis-like syndrome”.^24^, ^25^ Cytokines (e.g. Interleukin 6, Tumor necrosis factor α) and inflammatory markers, such as CRP and procalcitonin (PCT) are valuable in assessing the severity of post-cardiac arrest syndrome (PCAS). Elevated levels and their progression correlate with organ failure and poor outcomes. Although PCT and cytokines among other markers offer promise for predicting disease progression, they are not routinely measured and are not widely available, limiting their utility for early decision-making. However, according to our data, CRP as a routinely available acute-phase protein, may be useful for management and therapy control after an OHCA. Moreover, our analysis confirmed an excellent predictive power of CRP. In case of further validation, this suggests its inclusion in future prediction models and research on early laboratory parameters.

In contrast to the established rCAST score, our analysis did not find witnessed cardiac arrest or pH to be independent predictors. In addition, we did not analyze the motor score of the Glasgow coma scale (GCS) because it depends on the use and dosage of post-resuscitation sedatives. Thus, in our opinion GCS may not accurately reflect the patient's baseline condition. These mentioned aspects suggest the possibility of greater variance in these parameters, potentially affecting the validity of the rCAST score.

However, it has to be pointed out, that outcome after OHCA is complex and influenced by multiple factors, including patient characteristics and the quality of pre- and in-hospital management. In this context, it is critical to clearly define not only pre-hospital management but also in-hospital treatment standards at CACs to optimize outcomes.^26^ Moreover, various pathologies cause cardiac arrest and in turn different complications that need to be identified and managed at an early stage during hospitalization. The successful treatment of these complications depends on the type and severity of the complication but ultimately has an impact on patient outcome.

In the absence of definitive scientific data, the process of prognostication should be based on a multimodal approach using cumulative data from therapy, including imaging (e.g., CT scans), functional testing (e.g., electroencephalogram), electrophysiology, hemodynamics, clinical observations, and biomarkers.

In addition, the EPOS-OHCA model integrates parameters that reflect the underlying principles and pathophysiologic challenges after OHCA and in PCAS, such as severity of inflammation (CRP) and indicators of systemic perfusion and organ function (time to ROSC, initial rhythm, lactate, GFR).

The clinical application of this combination of parameters, considering their cut-off values, may therefore provide further valuable support for prognosis and decision-making in the often time-critical situation following an OHCA. This includes not only decisions to WLST but also the immediate initiation of appropriate treatments such as mechanical circulatory support to optimize hemodynamics and perfusion, targeted temperature management, and anti-inflammatory measures (e.g. immunoadsorption).

## Limitations

Several limitations need to be considered when interpreting the results of our study. First, this study includes a single-center design, which may limit the generalizability of our results and affect the reliability of the universal application of the risk scores and predictors. Second, due to the retrospective nature of the observation and data analysis, further hidden confounding factors are possible. This refers to the limited validity of our results due to missing data and the consecutive need to adjust the cohort size accordingly. Third, only data from non-traumatic OHCA patients are included in the analysis.

Risk assessments are nevertheless essential for the direction of research. They can provide valuable information about measures to be taken. However, since their assessment and application are not yet fully understood due to, among other things, the multimodality of the situation and pathophysiology after OHCA, this should be done with appropriate care. To further confirm the results, there is a need for external validation in different clinical settings and cohorts.

## Conclusions

In conclusion, the EPOS model is predictive of survival and favorable neurological outcome after non-traumatic OHCA in our single-center cohort. External validation is needed to determine whether the EPOS model can provide clinical decision support immediately after ROSC.

## Funding

No funding was received for conducting this study.

## Competing interests

JK received research funding from CytoSorbents; BM received research funding from Abiomed; JK, GC, BS, and BM receive speakers’ honoraria from Abiomed; JK and BM received speakers’ honoraria from Astra Zeneca, BS received speakers’ honoraria from Bayer and GSK. No other authors reported disclosures.

## CRediT authorship contribution statement

**Julian Kreutz:** Writing – review & editing, Writing – original draft, Visualization, Supervision, Formal analysis, Data curation, Conceptualization. **Nikolaos Patsalis:** Writing – review & editing, Writing – original draft, Formal analysis. **Charlotte Müller:** Investigation, Formal analysis, Data curation. **Georgios Chatzis:** Methodology, Formal analysis. **Styliani Syntila:** Writing – original draft, Visualization. **Kiarash Sassani:** Writing – original draft, Visualization, Formal analysis. **Susanne Betz:** Resources, Project administration, Data curation. **Bernhard Schieffer:** Writing – review & editing, Writing – original draft. **Birgit Markus:** Writing – review & editing, Writing – original draft, Supervision, Conceptualization.

## Declaration of competing interest

The authors declare the following financial interests/personal relationships which may be considered as potential competing interests: Julian Kreutz reports a relationship with Abiomed Europe GmbH that includes: speaking and lecture fees. Birgit Markus reports a relationship with Abiomed Europe GmbH that includes: funding grants and speaking and lecture fees. Georgios Chatzis reports a relationship with Abiomed Europe GmbH that includes: speaking and lecture fees. Bernhard Schieffer reports a relationship with Abiomed Europe GmbH that includes: speaking and lecture fees. Julian Kreutz reports a relationship with CytoSorbents Europe GmbH that includes: funding grants. Julian Kreutz reports a relationship with AstraZeneca that includes: speaking and lecture fees. Birgit Markus reports a relationship with AstraZeneca that includes: speaking and lecture fees. Bernhard Schieffer reports a relationship with Bayer AG that includes: speaking and lecture fees. Bernhard Schieffer reports a relationship with GSK that includes: speaking and lecture fees. If there are other authors, they declare that they have no known competing financial interests or personal relationships that could have appeared to influence the work reported in this paper.
