# Supplementary material for: EPOS-OHCA: Early Predictors of Outcome and Survival after non-traumatic Out-of-Hospital Cardiac Arrest
Source: Resusc Plus. 2024 Jul 24;19:100728. doi: 10.1016/j.resplu.2024.100728 (PMC11327594; doi:10.1016/j.resplu.2024.100728)
Supplement: Supplementary Data 1 [file mmc1.docx]

**Supplementary material**

**Suppl. Table 1.** **Exclusions from the overall cohort for further analysis (101 out of 564 patients).**

Abbreviations: ROSC: return of spontaneous circulation, EMS: emergency medical services.

|  | **n=101** |
| --- | --- |
| Never achieved ROSC | 82 |
| Traumatic cause of resuscitation | 8 |
| No resuscitation by EMS (bystander CPR only) | 4 |
| Missing data on resuscitation event | 7 |

**Suppl. Table 2. Causes of cardiac arrest.** ^1^: n (%).

| **Causes of cardiac arrest** | **non-survivors**  **n=246** | **survivors**  **n=217** | **p-value** |
| --- | --- | --- | --- |
| Cardiac event^1^ | 89 (36.2) | 142 (65.4) | <0.001 |
| Pulmonary artery embolism^1^ | 31 (12.6) | 7 (3.2) | <0.001 |
| Cerebral hemorrhage^1^ | 6 (2.4) | 3 (1.4) | 0.41 |
| Bolus death/ hypoxia^1^ | 55 (22.4) | 31 (14.3) | 0.03 |
| Drowning^1^ | 2 (0.8) | 1 (0.5) | 0.64 |
| Aortic dissection^1^ | 2 (0.8) | 1 (0.5) | 0.64 |
| Electrolyte imbalance^1^ | 4 (1.6) | 6 (2.8) | 0.40 |
| Intoxication^1^ | 2 (0.8) | 4 (1.8) | 0.33 |
| Other^1^ | 14 (5.7) | 9 (4.1) | 0.45 |
| Unknown cause^1^ | 41 (16.7) | 13 (6.0) | <0.001 |

**Suppl. Table 3: Multivariable regressions analysis (dependent variable: survived, Odds ratios (95 % CI).** Model 1 contains all variables that showed significant differences between survivors and non-survivors in the univariate analyses (categories: "Table 1: demographics and comorbidities", "Table 2: Pre-hospital resuscitation and baseline laboratory parameters (within the first hour after hospital admission"). After BMI was proved to be a non-informative predictor (OR, CI, p-value) in Model 1, the variable was excluded due to the large number of missing values (Model 2). Abbreviations: BMI: body mass index, VT: ventricular tachycardia, VF: ventricular fibrillation, CPR: cardiopulmonary resuscitation, CRP: C-reactive protein, GFR: glomerular filtration rate.

|  | **Model 1** | | **Model 2** | |
| --- | --- | --- | --- | --- |
| Number of valid data (n=) | 272 | | 398 | |
| Pseudo-R² (Cox&Snell; Nagelkerke) | 0.37; 0.50 | | 0.35; 0.47 | |
| AUC [95% CI] | 0.87 [0.83 – 0.91] | | 0.86 [0.82 – 0.89] | |
|  | **OR (95% CI)** | **p-Value** | **OR (95% CI)** | **p-Value** |
| **Demographics and comorbidities** |  |  |  |  |
| Age (years) | 0.98 (0.95 – 1.01) | 0.15 | 0.97 (0.95 – 0.99) | **0.002** |
| Male sex | 1.11 (0.51 – 2.40) | 0.79 | 0.88 (0.49 – 1.55) | 0.65 |
| BMI (kg/m^2^) | 0.98 (0.92 – 1.04) | 0.56 | --- | --- |
| Chronic renal failure KDIGO ≥ stage 3 (Ref.: No) | 0.52 (0.16 – 1.68) | 0.27 | 0.93 (0.37 – 2.29 | 0.87 |
| **Resuscitation parameters** |  |  |  |  |
| Initial shockable rhythm (VF/VT) (Ref.: No) | 3.03 (1.53 – 5.97) | **0.001** | 3.10 (1.81 – 5.32) | **<0.001** |
| Resuscitation time > 20 minutes | 0.27 (0.13 – 0.57) | **0.001** | 0.50 (0.28 – 0.88) | **0.02** |
| Mechanical CPR (chest compression device) | 0.64 (0.26 – 1.56) | 0.32 | 0.64 (0.28 – 1.45) | 0.28 |
| **Baseline Laboratory parameters** |  |  |  |  |
| pH | 0.75 (0.07 – 8.15) | 0.81 | 1.78 (0.28 – 11.27) | 0.54 |
| Lactate  (from arterial blood gas analysis) | 0.86 (0.78 – 0.95) | **0.003** | 0.87 (0.80 – 0.94) | **<0.001** |
| CRP | 0.90 (0.71 – 1.14) | 0.39 | 0.79 (0.66 – 0.94) | **0.01** |
| GFR | 1.02 (1.00 – 1.04) | **0.02** | 1.01 (1.00 – 1.03) | **0.04** |

**Suppl. Table 4. Cut-off values for predictors in the EPOS model using the Youden’s Index.** Abbreviations: ROSC: return of spontaneous circulation, CRP: C-reactive protein, GFR: glomerular filtration rate.

| **Demographics and comorbidities** | **Cut-off values** | **Sensitivity** | **1 - Specificity** |
| --- | --- | --- | --- |
| Age (years) | 67.5 | 0.54 | 0.40 |
| **Resuscitation parameters** |  |  |  |
| Resuscitation time until ROSC (min) | 18.5 | 0.67 | 0.41 |
| **Baseline laboratory parameters** |  |  |  |
| Lactate (mmol/l) | 8.2 | 0.71 | 0.18 |
| CRP (mg/l) | 12.0 | 0.46 | 0.20 |
| GFR (ml/min) | 60.5 | 0.50 | 0.19 |
